# Announcement of the Fifth Annual Session of the International Hahnemannian Association

**Published:** 1885-04

**Authors:** Rollin R. Gregg, J. B. Gregg Custis

**Affiliations:** President; Secretary


### ANNOUNCEMENT OF THE FIFTH ANNUAL SESSION OF THE INTERNATIONAL HAHNEMANNIAN ASSOCIATION.

The next annual session of the International Hahnemannian Association is called to convene at the Court-house, Syracuse, N. Y., on Tuesday, the 23d day of June, 1885, to continue three days. It is clearly the duty of every member of the Association to be present at that meeting and assist in the important work in hand; and it is felt to be the duty of every physician, whether member or not, who believes in the practice of pure Homœopathy and the preservation of the higher and better teachings of Hahnemann, to give the great cause the encouragement of his presence and voice in pleading for the better way.

Serious troubles, and sometimes great calamities, are absolutely necessary to enforce upon men an obedience to moral and scientific truths, to call back the wandering to an allegiance to principles, to arouse the lukewarm and doubting, to encourage those who are true, but discouraged at the long delay in the fruition of their highest and noblest hopes, and to compel the unbelieving and scoffing to halt in their course and consider that *law* reigns supreme in every department of nature, and *must be obeyed*, or the direst consequences must follow.

The entire civilized world stands to-day facing, is almost in the presence of, a most terrible impending calamity (judging by past history), in the threatened visitation, within the next two or three years, to every city, town, and hamlet of civilization, of the worst scourge of modern times, the true Asiatic cholera. But what encouragement has the dominant school of medicine, or any of its imitators in part or in whole, to offer in the treatment of this scourge, or to sustain us in facing such an evil?

Look to Marseilles and Toulon for your answer. *Seventy per cent.* of all attacked with cholera in those cities last summer and fall speedily found their graves, to say nothing of many in the small percentage saved who will inevitably date the beginning of serious chronic diseases to such attack, or to the suppressing treatment administered for it.

What hope, moreover, does the vaunted bacteria theory offer in this great emergency? Look again to Marseilles and Toulon for your answer. Koch, Pasteur, and other great bacteria lights on the ground, and yet *seventy per cent.* of all attacked died; or *just the same* percentage in the death-rate as half a century ago, when the disease appeared for the first time in Europe, and *when all were utterly ignorant of it.*

Truly, here is a proper place to call a halt and to take our bearings. Truly here the wanderer, the doubter, the discouraged, the scoffer, the unbeliever in the majesty of law, are all forced on to common ground, and are all compelled to work together for the common defense. But what shall that defense be? what must it be to compass the greatest security?

Look, now, to the immortal Hahnemann and to his equally immortal work. He it was who, guided by law (not simply by a "rule of practice," for he had had no experience in the treatment of this disease to formulate rules of practice upon), *but guided by law*, and without ever having seen a case of cholera, pointed out, under that unerring law, the true remedies which under ordinarily favorable conditions have SAVED *ninety per cent.*, often more, of all attacked; and under the *worst* conditions have never lost as high as thirty per cent., or *saved* seventy per cent. or over, under the most adverse circumstances, instead of having lost that percentage, as has allopathy under the best conditions. Where is the proof of all this? is it asked?

Examine the official statistics of past epidemics, as well as those of last year, for allopathy; and for Homœopathy see the statistics (also official) in *Joslin on Cholera,* and elsewhere. For Homœopathy, look at the official report of Admiral Mordvinow, President of the Imperial Council of Russia, for the epidemic of 1831–32: "Not a single death has occurred where homœopathic treatment was resorted to in the incipient symptoms of the cholera;" and "All the patients cured by Homœopathy regained in a very short time their former health and strength, while those who survived other treatments were left in a state of weakness which lasted several months, and but too often terminated in another disease which proved fatal."

Look, also, at the report of Madam Lvoff, "of the Government of Saratow," for Homœopathy: "Four hundred cholera patients saved and restored to perfect health. * * * Fifty patients in our own village, and not *one* of them *died*. * * * All the sick who took medicine in strict conformity to the rules were saved, although some of them were already in the state of collapse, which apparently precluded all hope. In this last stage there were not a few with their teeth clenched so fast that it was necessary to force them open for the purpose of introducing the medicine, and yet, on the very day following, they were relieved and convalescent." And these grand results, be it understood, were not accomplished even by doctors, but by intelligent laymen under the guidance of *law*.

Look at the three hundred and seventy-seven cases, *without a single death*, treated by one homœopathic physician in a former epidemic in Naples, where, also, last year, seventy per cent. were lost under allopathy. Look at the success of the venerable Rev. Dr. Weith, of Vienna, not a physician—one hundred and twenty-five cases treated and but three deaths. Look at the results for Homœopathy in the hands of the late Dr. Pulte, of Cincinnati, and of Dr. Benjamin Ehrman, member of our Society, in the treatment of the scourge in that city in 1849. Eleven hundred and sixteen cases of cholera treated with a loss of only three per cent., and thirteen hundred and fifty cases of cholerine treated and not a death. Is this not enough?

Diverging here to another most prominent so-called bacterial disease, viz.: diphtheria, what do we find? Look this time to New York city—one of the great centres for medical wisdom for this country and even for the world—for your answer, and what is that? Nearly *two-thirds* of all reported cases of diphtheria that occurred in that city this last fall and winter died! And yet it is here asserted, without fear of contradiction by the results of practice, that at least *ninety per cent.* of all cases of diphtheria can be saved by the purest practice of Homœopathy.

Ninety per cent. or more of all cases of cholera, ninety per cent. or more of all cases of diphtheria, *saved*, by pure homœopathic practice, under the law, as against fifty to seventy per cent.

*lost* under all other methods of treatment in violation of law. Here, surely, is something worth working for, worth combining for, worth praying for, yea, indeed, worth *fighting* for, if need be for the redemption of man from the thralldom of such terrible diseases.

But, it may be said, there are other societies with the same high purposes, and the same aims for the advancement of Homœopathy, then why establish another and rival society, to arouse contentions and weaken our forces? *Is this true?* Is it true that there is another society in all this wide world, excepting the Lippe Society, of Philadelphia, and the Central New York Homœopathic Society at the home of which we are to meet, that is working at all to spread the pure practice of Homœopathy and to enforce the principles of Hahnemann in their highest practical application? Is there another society where the clinical reports of cures wrought (which is *the only possible test* of the *curative* powers of medicine), yea, of brilliant cures wrought under the guidance of the best teachings of Hahnemann, are welcome and properly treated? To these questions we may all find the correct answer in the certified and published "proceedings" of those societies, and what is it? Much of those proceedings, sustained by the utterances of the majority of their members, *proves* that their increasing effort has been for years, and growing bolder in it every year, to induce a belief that we have no law to guide us, but only "a rule of practice" that may be violated at will, and to thereby drag down the high standard of Homœopathy from the lofty eminence where Hahnemann placed and left it; and all for what? Why, to secure "liberty of medical opinion and action" to adopt, in part at least, the ways and methods of, if not to force a ruinous affiliation with, that school of medical practice which showed the best work it is capable of doing in the stricken cities of France last year—in a death-rate of seventy per cent. where ten to fifteen per cent. should have been the extreme of losses. Do you want any more of that?

We may also look in another direction for an answer to our questions, and for the proof of the baleful influence of those societies against the highest interests of Homœopathy. Three-fourths, if not nine-tenths or more, of all young physicians graduated during the last ten or fifteen years, and sent out to practice Homœopathy, have no confidence whatever in the highest, purest, and truest teachings of Hahnemann, and are often found apologizing for or vigorously declaring their unbelief in them—sometimes, indeed, if not often, are found vying with their allopathic neighbors and friends in scoffing at and denouncing those teachings and all who believe in them. And why is all this? Not, certainly, because those teachings have been found misleading and inapplicable in practice, as the unexampled cholera statistics cited, and thousands of other proofs could be given to show; but it is because those young physicians have read the proceedings of the societies indicated; it is because they have heard the scoffings at such teachings by many of the so-called leaders in those societies; and, for very shame be it said, because they have heard it often asserted by those to whom they have looked for good counsel, that "Hahnemann was in his dotage," was "drunk with mysticism," etc., when he did the grandest work for man that man ever did, in his masterly work upon chronic diseases. What an outrage upon truth and decency; and this, too, where the highest and purest interests of all humanity are at stake! Why, Hahnemann was nearly eighty years old when he pointed out the true remedies for cholera that have already done so much for mankind—infinitely more than all his traducers ever have or ever will do. What a pity that those traducers could not have caught, in youth or middle age, a hundredth part of the "dotage" and "mysticism" that actuated him!

Can it be supposed for an instant that the young physicians referred to are the best prepared that they can be to grapple with the impending scourge when it does strike them? Is their possession of a hypodermic syringe, and their evident readiness to resort to Opium, Alcohol, Quinine, germicides, *et seq.*, and *a la* old school, under the first trying emergency, the best armamentarium that they can have to enter the fight with? Let the sad story of last year in Europe, and the history of the triumphant success of Homœopathy in all past epidemics of the scourge, give them their answer.

These young physicians, and those who have brought them to the state of mind which they are in, together with the families of both, are just as liable to the scourge—will, perhaps, be more so, on account of greater exposure than most others, and let them take warning in time. To just the extent that they trust in or adopt any portion of allopathy, to just that extent disaster and ruin await them.

True disciples of Hahnemann! A great duty is upon you. Are you equal to it? There has been a determined effort for fifteen or twenty years by a large portion of those who claim to be of our school to ruin our standing as a distinct school of medicine; and, whether intended or not, to deprive suffering humanity of the high hopes and encouragement which we can *honestly* and *justly* offer them in the treatment of the worst human maladies.

It is yours now, with abundant official statistics to aid you, to stay and counteract this deliberate process of ruin. Will you do it? Let the frightful story of Southern Europe last year, and the sublime record of Homœopathy in the treatment of cholera in all past epidemics, be your incentives to ACTION.

ROLLIN R. GREGG, M. D., *President.*
J. B. GREGG CUSTIS, M. D., *Secretary.*

